# Determination of the volatile and polyphenol constituents and the antimicrobial, antioxidant, and tyrosinase inhibitory activities of the bioactive compounds from the by-product of *Rosa rugosa Thunb. var. plena Regal* tea

**DOI:** 10.1186/s12906-018-2374-7

**Published:** 2018-11-20

**Authors:** Guixing Ren, Peng Xue, Xiaoyan Sun, Gang Zhao

**Affiliations:** 1 College of Pharmacy and Biological Engineering, Chengdu University, Chengdu, 610000 People’s Republic of China; 2 Social Risk Prediction and Management, School of Public Health and Management, Weifang Medical University, No.7166 Baotong West Street Weicheng District, Weifang, 261053 People’s Republic of China; 3 Key Laboratory of Coarse Cereal Processing, Ministry of Agriculture, No.2025 Chengluo Road, Longquanyi District, Chengdu, 610106 People’s Republic of China; 4 Institute of Crop Sciences, Chinese Academy of Agricultural Sciences, No.80 XUEYUAN South Road, Handian District, Beijing, 100081 People’s Republic of China

**Keywords:** RFCS, Phytochemical constituents, Antioxidant, Antimicrobial, Tyrosinase inhibitory activities

## Abstract

**Background:**

The phytochemical constituents and biological activities of *Rosa rugosa Thunb. var. plena Regal* flower cell sap (RFCS) were investigated.

**Methods:**

Volatile constituent, such as linalool, phenylethyl alcohol, citronellol, α-bisabolol, were identified by GC-MS. The contents of hyperoside, kaempferol-3-O-rutinosid, rutin, and luteolin as well as the total flavonoid content in RFCS were determined by HPLC and HPLC-MS. The total polyphenol content was evaluated by the Folin-Ciocalteu colorimetric method. The antioxidant activities of RFCS and the standards were evaluated by DPPH and ABTS radical scavenging assays. The tyrosinase inhibitory activities of the rose samples and standard substance were determined by a spectrophotometric method. The antimicrobial effects of RFCS were evaluated in terms of minimum inhibitory concentrations (MICs) and minimum bactericidal concentrations (MBCs) or minimum Fungicidal concentrations (MFCs).

**Results:**

The rose fraction exhibited a high content of biologically active ingredients. The total content of volatile compounds in RFCS was approximately 48.21 ± 2.76 ng/mL. The total phenolic acid content and total flavonoid content were 0.31 ± 0.01 mg/mL and 0.43 ± 0.01 mg/mL, respectively. Its IC_50_ value in the DPPH assay was 1120 ± 42 μg/mL, and its IC_50_ value for ABTS radical scavenging activity was 1430 ± 42 μg/mL.RFCS strongly inhibited L-tyrosine oxidation with an IC_50_ value of 570 ± 21 μg/mL. Every compound identified in RFCS exhibited broad-spectrum antimicrobial activity. *F. nucleatum* was most susceptible to RFCS with an MIC of 64 μg/mL and MBC of 250 μg/mL.

**Conclusions:**

Due to its rose-like aroma, phenylethyl alcohol may be combined with linalool for use as a natural skin-whitening agent and skin care additive in the and pharmaceutical industries.

## Background

The flower of *Rosa rugosa Thunb. var. plena Regal* is not only used in perfume production but has also been used as a health food and medicine in Asian countries for thousands of years. Additionally, roses contain active materials, such as essential oils, polyphenols, flavonoids and anthocyanin, which are known for their antimicrobial, anti-inflammatory, hypoglycemic, and antioxidant activities [1–4]. Roses can be consumed in many forms, such as rose teas, rose cookies, and rose oils. The production of rose tea or dried flower petals via low-temperature drying of rose flowers (*Rosa rugosa cv. Plena*) yields a condensate called “rose flower cell sap” (RFCS). The disposal of RFCS represents a great waste of resources due to its high content of polyphenols and rose essential oil, which has very high biological activity. In addition, environmental pollution may be caused by the improper disposal of RFCS because it is difficult to decompose. In addition, essential oils and polyphenols are active ingredients in the pharmaceutical, cosmetic, and food industries. The drying of 1 kg of raw rose petals or flower bud material can produce approximately 0.2 L of condensate. Approximately 40,000 kg of rose flower buds and 20,000 kg of petals are used per cycle of industrial microwave-drying in Pingyin alone. To date, no studies have reported a suitable method for the disposal of RFCS and the bioactive compounds contained therein.

Rose oil distillation wastewater (RODW) is another by-product of the steam distillation of dried rose flowers to product rose oil. In previous studies, RODW has been concentrated to generate a polyphenol-enriched residue containing non-volatile phenolic compounds [5]. Moreover, the polyphenol fraction of RODW can strongly inhibit mushroom tyrosinase (IC_50_ value of 0.41 μg/mL) [6]. Thus, the polyphenols in RODW may be used as a bioactive substance to relieve hyperpigmentation.

Food-related pathogenic bacteria cause foodborne illnesses in millions of people and even hundreds of deaths every year in the USA alone, and the associated cost total approximately $ 2.4 billion [7]. Thus, the increasing demand for healthy, non-toxic, and effective antimicrobial agents has inspired research on multifunctional, naturally produced food additives. Although rose oil primarily contains essential oils known for their antimicrobial activities [8], the antimicrobial effects of RFCS have not been investigated.

The phenolic compounds and volatile substances in flowers have strong biological activities such as antioxidant and tyrosinase inhibitory effects [9]. The development of additional methods for inhibiting tyrosinase activity is an active area of research in the functional cosmetics and food industries due to tyrosinase’s whitening effect and ability to control browning [10, 11]. Antioxidants may lower the risks of health concerns such as cancer, aging, and atherosclerosis by reducing the level of reactive oxygen species (ROS) [12]. Some antioxidants, such as ascorbic acid, also have been reported to have whitening effects [11].

In our preliminary test, antimicrobial, antioxidant, and tyrosinase inhibitory activities of RODW from *Rosa rugosa Thunb. var. plena Regal* were evaluated [13]. However, there are no reports in the literature investigating the phytochemical composition and biological activities of RFCS from *Rosa rugosa cv. Plena*. In this study, (1) contents of the total phenolics, flavonoids, total solid and volatile contents were investigated; (2) the antibacterial (six strains) and anti fungal (one strain) activity, antioxidant, and tyrosinase inhibitory activities of each active compound and RFCS were examined. Our results will help to improve the value of roses in the fields of medicinal and cosmetic products [13–16].

## Methods

### Chemicals

Phenylethyl alcohol, α-bisabolol, α-terpineol, citronellol, miconazole nitrate, hydrochloride tetracycline, menthol and camphor were purchased from J&K Scientific Ltd. (Beijing). Kojic acid, hyperoside, quercetin, gallic acid, kaempferol-3-O-acetylglucosylrhamnoside and kaempferol-3-O-glucoside were purchased from Sigma (Shanghai, China). Anaerobic blood agar base medium (CDC), actinomycete broth medium (GAM broth), brain heart infusion (BHI) broth, and nutrient agar were obtained from Suolaibao Biotech Co., Ltd. (Beijing, China). The remaining chemicals were analytical or chromatographic grade.

### Sample preparation

RFCS of *Rosa rugosa Thunb. var. plena Regal* was obtained from Fragrant Rose Biological Technology Co., LTD in Pingyin. The samples were filtered through a 0.42 μm microfiltration membrane prior to analyses. The total solid content of RFCS was evaluated by freeze-drying. The identification of *Rosa rugosa Thunb. var. plena Regal* was identificated by senior agronomist Guo and confirmed in voucher sample (Ser. No. 0712) deposited at Herbarium, Pingyin Institute of Rose Sciences.

### HPLC analyses

The concentration of polyphenol constituents in the extract was determined by HPLC and UV analyses. The HPLC apparatus was an LC-20A HPLC system (Shimadzu Corporation, Kyoto, Japan), and it was equipped with an Ultrasphere 5 C_18_ column (4.6 mm × 250 mm, Ultrasphere Co., Ltd., Berkshire, UK). The mobile phase was a gradient elution of water (A) and acetonitrile (B) and was programmed as follows: starting with 10% B for 10 min, 10–25% B between 15 and 20 min, 25–30% B between 20 and 25 min, 30–60% B between 25 and 50 min, 60–10% B between 50 and 51 min, and 10% B between 51 and 55 min. The flow rate of the mobile phase was maintained at 1 mL/min, the detector wavelength was set at 350 nm, the column oven was set at 25 °C, and the sample injection volume was 10 μL.

### HPLC-ESI-MS conditions

The electrospray ionization (ESI) mass spectrometry (MS) data were recorded on an Agilent-LC-1100 instrument (Agilent, USA). The HPLC conditions for the HPLC-ESI-MS analysis were as described above. The ESI parameters were as follows: the collision gas (N_2_) flow rate was maintained at 10 mL/min, the column oven was 25 °C, data were acquired in negative ionmode [M-H]^−^, scans were conducted over m/z 50–2000, the spray voltage was 4.5 kV, the capillary voltage was 10 V, and the capillary temperature was 250 °C. The components in the sample were identified based on their mass spectral data and retention time.

### GC/MS analysis

The volatile constituents in RFCS were determined by a Shimadzu GC/MS model QP2010 Ultra system equipped with an Rtx-5MS (30 m × 0.25 mm, film thickness 0.25 μm) capillary column. The oven program was as follows: starting at 60 °C, heating to 120 °C at a rate of 1.7 °C/min, heating to 200 °C at 2.5 °C/min, heating to 260 °C at a rate of 8 °C/min, and finally holding at 260 °C for 2 min. Helium was used as the carrier gas, and the flow rate was 1.0 mL/min. The injector and detector temperatures were held at 250 °C and 280 °C, respectively. A split injection was conducted in splitless mode. The ion source temperature was 250 °C and its ionization energy was 70 eV. The mass range was 35–500 Da. The components in the sample were identified based on their mass spectral data and retention time.

### Preparation of standard curves

Solutions of phenylethyl alcohol (2.23 mg), α-bisabolol (2.1 mg), α-terpineol (5.23 mg), citronellol (1.52 mg), menthol (1.32 mg), and camphor were separately prepared in 1 mL of acetonitrile. Next, the stock solutions were diluted by factors of ten thousand to one billion with ethyl acetate, and 1 μL of each sample was analyzed by GS/MS. Solutions of kojic acid (1.12 mg), hyperoside (1.07 mg), quercetin (1.07 mg), gallic acid (1.29 mg), and kaempferol-3-O-acetylglucosylrhamnoside (1.15 mg) were separately prepared in 1 mL of methyl alcohol. The stock solutions were diluted by factors of 2 with methyl alcohol, and 10 μL of each solution was analyzed by HPLC. Each concentration of working solution was analyzed in three times. The calibration curves were plotted as the peak areas against the concentration of each standard. The content of the reference substance in each sample was calculated using the calibration curves.

### Determination of total phenolics, flavonoids and total solid content

The total phenolic contents of the RFCS were evaluated by the Folin-Ciocalteu colorimetric method [17]. The total content of phenolic substance was determined by comparison to a standard curve of gallic acid. The total flavonoid contents of the RFCS samples were evaluated by HPLC, which provided the total amount of all tested flavonoid compounds. A 10-mL sample of RFCS was freeze-dried to determine the total solid content. Every determination was carried out in triplicate.

### Antioxidant properties

#### DPPH radical scavenging activity

The antioxidant activity of RFCS and the standards were evaluated by DPPH radical scavenging activity using a slightly modified version of a previously reported method [18]. Briefly, 10 μL aliquots of the rose samples (1000 μg/mL to 62.5 μg/mL) were mixed with 190 μL of 50% ethanol containing 0.4 mM DPPH and incubated in the dark for 30 min. Aliquots (100 μL) of the supernatants were transferred into a 96-well microplate, and the absorbance of each was recorded at 517 nm using a Spectramax Plus384 UV-Vis spectrophotometer (Molecular Devices, Sunnyvale, California, USA). Ascorbic acid (1000 μg/mL to 0.05 μg/mL) was used as a positive control, and DPPH solution without sample was used as the negative control. The IC_50_ values, which represent the concentrations of rose samples and standard substance at which 50% of the DPPH radical was inhibited, were determined. The tests were performed in triplicate, and the percentage of DPPH scavenging was calculated using the following equation.

$$ \mathrm{Inhibition}\ \left(\%\right)=\left\{\left({\mathrm{H}}_0-\mathrm{H}\right)/{\mathrm{H}}_0\right\}\times 100 $$

$$ \mathrm{H}:\mathrm{Absorbance}\ \mathrm{of}\ \mathrm{RFCS}\ \mathrm{and}\ \mathrm{the}\ \mathrm{standards};{\mathrm{H}}_0:\mathrm{Absorbance}\ \mathrm{of}\ \mathrm{the}\ \mathrm{blank} $$

#### Determination of ABTS radical scavenging

The ABTS assay of RFCS was performed according to a modified version of a previously reported method [19]. Briefly, the stock solutions were generated by mixing equal quantities of 7.4 mM ABTS^●+^ solution and 2.6 mM potassium persulfate solution, and the mixture was incubated at room temperature for 12 h in the dark. Then, the solution was equilibrated with 1 mL of ABTS^●+^ solution with 50% ethanol serving as a positive control. The absorbance of the solution at 734 nm was 1.17 ± 0.02 units. Aliquots (10 μL) of the rose samples (1000 μg/mL to 62.5 μg/mL) were mixed with 1.0 mL of the diluted ABTS^•+^ solution. The mixture was mixed vigorously and incubated at 30 °C for 30 min. The absorbance was then measured at 520 nm with an excitation wavelength of 734 nm using the spectrophotometer. The positive standard was Trolox (2000 μg/mL to 0.05 μg/mL).

$$ \mathrm{Inhibition}\ \left(\%\right)=\left\{\left(\mathrm{Absorbance}\ \mathrm{of}\ \mathrm{blank}-\mathrm{Absorbance}\ \mathrm{of}\ \mathrm{sample}\right)/\mathrm{Absorbance}\ \mathrm{of}\ \mathrm{blank}\right\}\times 100 $$

### Determination of the Tyrosinase inhibitory activity

The tyrosinase inhibitory activities of the rose samples and standard substance were determined by a spectrophotometric method [17]. First, 300 μL aliquots of different concentrations (1000 μg/mL to 62.5 μg/mL) of each sample were diluted with 700 μL of 0.175 M sodium phosphate buffer (pH 6.8), then 1.0 mL of 10 mM DOPA solution and 1.0 mL of mushroom tyrosinase (220 units/mL) were added. Ethanol (300 μL, 50%) and kojic acid (2000 μg/mL to 0.1 μg/mL) were used as the blank reference and positive standard, respectively. The reaction mixture was vortexed and maintained at 37 °C for 15 min, and then the absorption maximum of dopachrome (set at 479 nm) was measured using a microplate reader (Molecular Devices, Sunnyvale, California, USA). The tests were performed in triplicate, and the value of tyrosinase inhibition activity was calculated as described above.

### Antimicrobial properties

#### Antibacterial and antifungal assays

The antimicrobial activity was measured by the method described by Xue [20]. All standard strains were obtained from the Guangdong Microbiology Culture Center (Guangzhou, China). *Listeria ivanovii* (ATCC 19119) was cultured in BHI, *Salmonella enteritidis enteritidis* (ATCC 14028) *Staphylococcus aureus* (ATCC 25923) and *Escherichia coli* (ATCC 25922) were cultured in nutrient agar (NA) for 24 h and at 37 °*C. candida albicans* (ATCC 10231) was cultured in PHB at 37 °C for 24 h. *Propionibacterium acnes* (ATCC 6919) and *Fusobacterium nucleatum* (ATCC 10953) were cultured in CDC agar at 37 °C for 48 h in a YQX-II anaerobic incubator (Shanghai, China). The final cell counts in l mL of broth were approximately 10^6^ colony-forming units (CFU/mL). A 10 mg/mL solution of miconazole nitrate and hydrochloride tetracycline in water was used as a positive control against fungi and bacteria, respectly.

#### Determination of minimum inhibitory concentration (MIC) and minimum bactericidal concentration (MBC) or minimum fungicidal concentration (MFC)

The MIC and MBC or MFC values were determined as described previously by Xue. Briefly, 100 μL dilutions (approximately 100,000 CFU/mL) of *Staphylococcus aureus*, *Escherichia coli*, *Salmonella enteritidis enteritidis*, *Fusobacterium nucleatum*, and *Candida albicans* in nutrient broth and *Listeria ivanovii* and *Propionibacterium acnes* in GMA broth were inoculated into microtiter plates. Then, 100 μL aliquots of the solutions of the test compound were added after a two-fold serial dilution with nutrient broth (from 2 mg/mL to 3 μg/mL). Broths with 5% (*v*/v) DMSO were used as controls. The petri dishes were incubated at 37 °C for 24 h with the exception of *Propionibacterium acnes* and *Fusobacterium nucleatum*, which were incubated at 37 °C for 48 h. The MIC was recorded as the lowest concentration of sample showing no detectable growth. To determine the MBC or MFC values for no bacterial or fungus growth, 10 μL of sub-inhibitory concentrations of the test compounds were incubated on CDC or GMA agar plates for 24 or 48 h. Every determination was carried out in triplicate.

### Date analysis

Data are presented as the mean of three replicates ± standard deviation. One-way ANOVA with Duncan’s multiple range test was used to analyze the results with SPSS 13.0 and Sigma Plot 10.0, respectively, using a computer (Lenovo, Yangtian B 41) equipped with the Win 7 operating system. A *p* value of < 0.05 was determined to be statistically significant.

## Results and discussion

### The contents of volatile substance

Since the RFCS samples had a specific rose fragrance, we analyzed and compared the volatile components of its ethyl acetate extract. The contents of the volatile components of the ethyl acetate extract of RFCS were determined by GC/MS and analyzed by comparison to four standard curves, and the results are expressed as ng/mL.

**Fig. 1:**
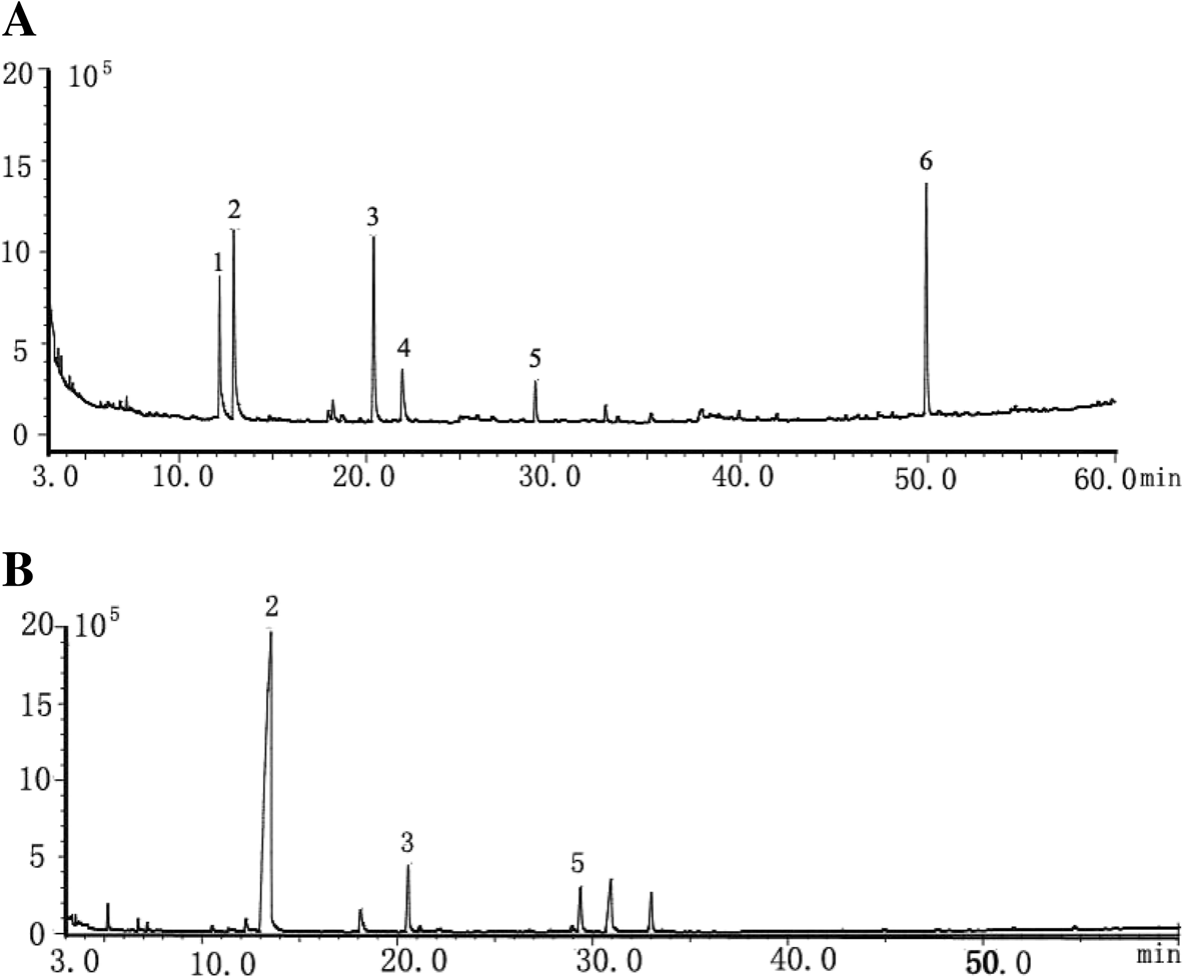
**a** Total ion chromatogram of volatile compounds in ethyl acetate from standard substance (**a**), and rose flowers cell sap (**b**). RFCS: rose flower cell sap. Identification of peaks. 1, linalool; 2, phenylethyl alcohol; 3, citronellol; 4, ester phenylethyl acetate; 5, citronellol acetate; 6, α-bisabolol

Six principal components were simultaneously identified according to their standard retention times and MS ion fragments. The GC chromatograms of the reference substance in RFCS are shown in Fig. 1. The content of each element in every sample is presented in Table 1. As shown in Fig. 1, six compounds were successfully separated under the gradient temperature program. The total content of volatile compounds in RFCS was approximately 48.21 ± 2.76 ng/mL, and six major kinds of volatile compounds, including phenylethyl alcohol (40.48 ± 2.24 ng/mL), citronellol (7.83 ± 0.77 ng/mL), α-bisabolol (0.08 ± 0.01 ng/mL) and phenylethyl acetate (11.20 ± 0.89 ng/mL) were identified (two peaks have not been identified and the content of linalool is rare). In previous studies on the volatile compounds in RODW, GC-MS, more specifically HS-SPME/GC/MS, techniques have been widely utilized [13, 21–24]. In contrast to these previous studies, our study reports the absolute contents of the components. Although there was a broad range of volatile compounds, there were no differences in the dominant components. The major volatile compounds in RFCS were monoterpene alcohols (citronellol, linalool, and phenylethyl alcohol, which is specific to small roses). The types of dominant components in RFCS are similar to those of RODW, but there is a significant difference in the contents of the components [13]. One possible reason for these differences is that most of the volatile components were lost in the rose tea drying process.

**Table 1:** Analytical characteristics of volatile substances in rose waste. (ng/mL)

Compound	RT	Formula structure	Weight	RFCS
Linalool	12.163	C_10_H_18_O	154.25	n.d
Phenylethyl alcohol	12.921	C_8_H_10_O	122.16	40.48 ± 2.24
Citronellol	20.393	C_10_H_20_O	156.27	7.83 ± 0.77
Phenylethyl acetate	21.923	C_10_H_12_O_2_	164.2	11.20 ± 0.89
Citronellol acetate	29.029	C_12_H_22_O_2_	198.3	n.d
α-bisabolol	49.909	C_15_H_26_O	222.36	0.08 ± 0.01
Total content				48.21 ± 2.76

*nd* not detected; Data are expressed as mean ± standard deviation of triplicate samples; *RFCS* rose flower cell sap

### Total phenolic, flavonoid and soild contents

**Fig. 2:**
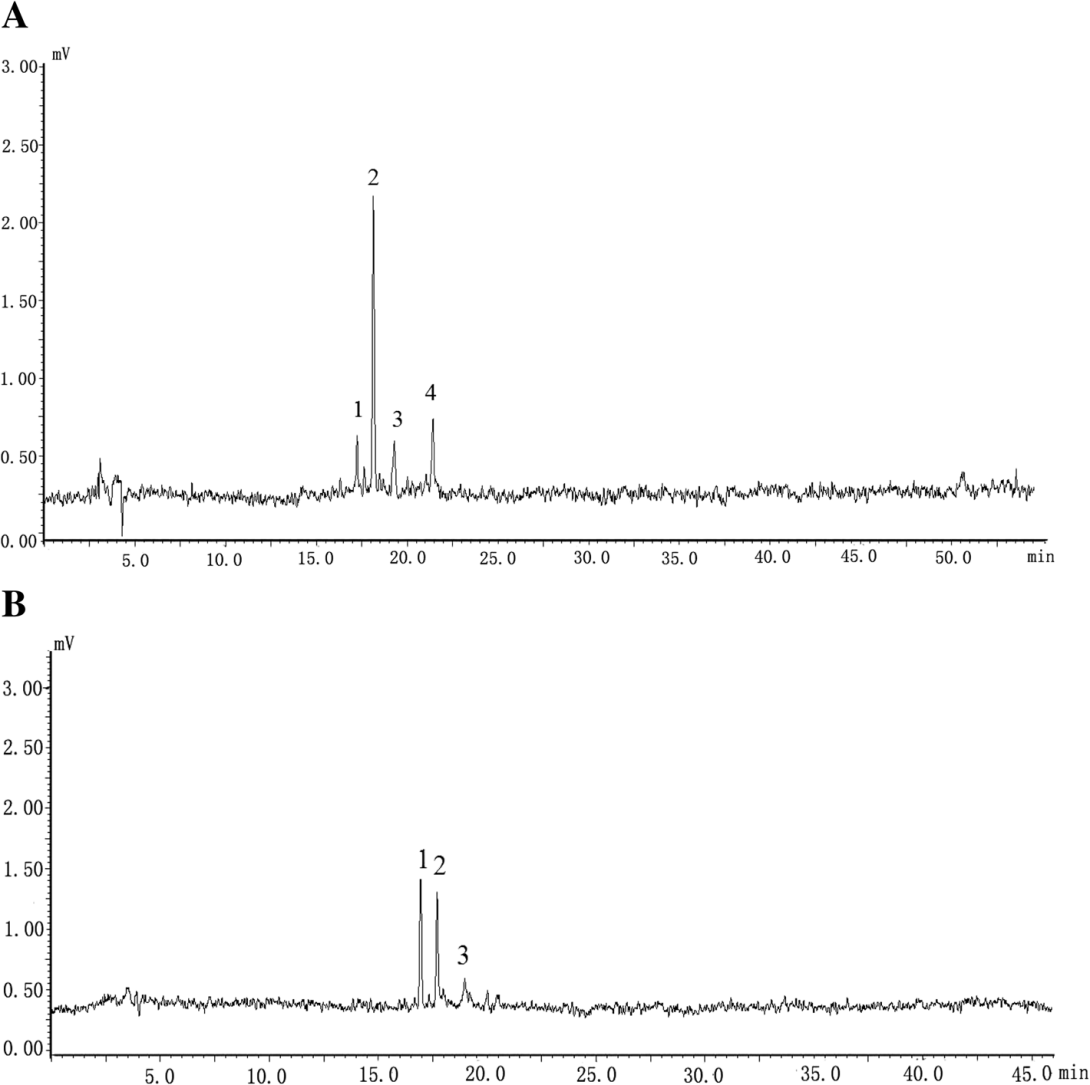
HPLC chromatogram of standard substanceb (**a**), and rose flower cell sap (**b**). RFCS: rose flowers cell sap. Identification of peaks: 1, hyperoside; 2, kaempferol-3-O-rutinoside; 3, rutin; 4, luteolin

The flavonoids, their retention times, and the calibration curves of standard compounds in RFCS, as determined using HPLC, are showen in Fig. 2. Four compounds were successfully separated under the gradient temperature program, as showen in Fig. 2. The linearity of calibration curves and regression coefficients of flavonoids were demonstrated in Table 2. It was found that the reference compounds showed good linearity (R^2^ ≥ 0.997). RFCS was found to contain three main components, namely, hyperoside (0.18 ± 0.01 mg/mL), kaempferol-3-O-rutinosid (0.12 ± 0.01 mg/mL), and rutin (0.23 ± 0.01 mg/mL). The total phenolic content and total flavonoid content were 0.31 ± 0.01 mg/mL and 0.43 ± 0.01 mg/mL, respectively. The total solid content in RFCS was 1.45 ± 0.04 mg/mL.

**Table 2:** Analytical characteristics of compounds in rose waste. (mg/mL)

Peak	Ginsenoside	Retention time	Calibration curve	R^2^	RFCS
1	hyperoside	16.981	y = 14,955x-5214	0.9989	0.18 ± 0.01
2	kaempferol-3-O-rutinoside	17.737	y = 7102x + 3256	0.9984	0.12 ± 0.01
3	rutin	19.01	y = 41,285x-43,792	0.9979	0.23 ± 0.01
4	luteolin	22.01	y = 31,527x-5241	0.9991	n.d
	total phenolic content				0.31 ± 0.01
	total flavonoid content				0.43 ± 0.01
	total solid content				1.45 ± 0.04

*nd* not detected; Data are expressed as mean ± standard deviation of triplicate samples; *RFCS* rose flower cell sap

Previous studies have reported that the dominant phenolic and flavonoid compounds in rose rugosa tea were gallic acid, catechin, epicatechin, and quercetin and the total polyphenol content and flavonoid content in the *Rosa rugosa* tea polyphenol extract were 875.2 mg/g and 610.3 mg/g, respectively [1]. In addition, rutin, multiflorin B, hyperoside, kaepferol, and ellagic acid were also found in the resin fractions of RODW [6]. Furthermore, unlike previous studies, although our study used HPLC-MS to determine the phenol and flavonoid compounds in RODW, only one of the dominant compounds, kaempferol-3-O-rutinosid, was found in this study and in previous studies [6, 13]. Neither of the phenol compounds was detected in RFCS by HPLC primarily because the concentrations of phenols and flavonoids in RFCS are very low, and thus they are undetectable by HPLC. Those solids in RFCS were mixture of small molecules.

### Antioxidant capacity

Table 3 presents the DPPH IC_50_ values of RFCS and the standard compounds. The flavonoids with IC_50_ values < 1 μg/mL, including hyperoside (IC_50_ value of 0.695 ± 0.021 μg/mL), kaempferol-3-O-rutinoside (IC_50_ value of 0.808 ± 0.024 μg/mL), rutin (IC_50_ value of 0.715 ± 0.017 μg/mL), and luteolin (IC_50_ value of 0.507 ± 0.015 μg/mL), showed stronger DPPH radical scavenging activities than RFCS (IC_50_ value of 1120 ± 42 μg/mL). Single volatile compounds, such as linalool, phenylethyl alcohol, citronellol, and α-bisabolol, showed weak radical scavenging activity with IC_50_values of > 10,000 μg/mL. In previous reports, the antioxidant activities of various natural products, including those from rose, have been attributed to the contents of phenolic compounds [25, 26]. The ABTS radical assay is also used to evaluate the radical scavenging activity of hydrogen-donating and chain-breaking antioxidants in many natural products [27, 28]. As shown in Table. 3, the ABTS radical scavenging activities of single compounds and RFCS are expressed as μg/mL. Consistent with previous works, flavonoids exhibited significantly higher antiradical activities and antioxidant capacities than volatile compounds [8, 13]. In the present study, the results of ABTS scavenging were similar to those of DPPH; flavonoid compounds with IC_50_ values < 1 μg/mL, including hyperoside (IC_50_ value of 0.526 ± 0.014 μg/mL), kaempferol-3-O-rutinoside (IC_50_ value of 0.719 ± 0.016 μg/mL), rutin (IC_50_ value of 0.621 ± 0.024 μg/mL), and luteolin (IC_50_ value of 0.436 ± 0.026 μg/mL), showed stronger ABTS radical scavenging activities than RFCS (IC_50_ value of 1430 ± 49 μg/mL). Single volatile compounds, such as linalool, phenylethyl alcohol, citronellol, and α-bisabolol, exhibited weak antiradical activities (IC_50_ values of > 10,000 μg/mL).

**Table 3:** Total solid content and IC_50_ values of single compounds in rose products. (μg/mL)

	DPPH radicalscavenging activity	Tyrosinase inhibition	ABTS radicalscavenging activity
RFCS	1120 ± 42 b	570 ± 21 ab	1430 ± 49 b
Linalool	> 10,000 a	730 ± 44 a	> 10,000 a
Phenylethyl alcohol	> 10,000 a	315 ± 13 b	> 10,000 a
Citronellol	> 10000a	825 ± 31 a	> 10,000 a
α-bisabolol	> 10,000 a	635 ± 22 a	> 10,000 a
Hyperoside	0.695 ± 0.021 c	0.762 ± 0.018 d	0.526 ± 0.014 c
Kaempferol-3-O-Rutinoside	0.808 ± 0.024 c	0.908 ± 0.021 d	0.719 ± 0.016 c
Rutin	0.715 ± 0.017 c	0.856 ± 0.014 d	0.621 ± 0.024 c
Luteolin	0.507 ± 0.015 c	0.613 ± 0.016 d	0.436 ± 0.026 c
Positive control	0.449 ± 0.013 c	80 ± 17 c	0.324 ± 0.019 c

Data are expressed as mean ± standard deviation of triplicate samples; *RFCS* rose flower cell sap

Values in each column followed by different letters are significantly different (*P* < 0.01)

### Tyrosinase inhibitory activities

Tyrosinase is a multifunctional copper-containing enzyme found in fungi, mammals, and plants [29]. Tyrosinase has two distinct enzyme activities, namely, monophenolase activity and diphenolase activity [30]. We conducted an initial study of the tyrosinase inhibitory activities of mushroom tyrosinase. As per this assay, RFCS showed strongly tyrosinase inhibitory activities with an IC_50_ value of 570 ± 21 μg/mL (Table 3). The volatile compounds, including linalool, phenylethyl alcohol, citronellol, and α-bisabolol, also showed dose-dependent tyrosinase inhibitory effects with IC_50_ values of 730 ± 44 μg/mL, 315 ± 13 μg/mL, 825 ± 31 μg/mL, and 635 ± 22 μg/mL, respectively. All the flavonoid compounds, namely, hyperoside, kaempferol-3-O-rutinosid, and even rutin were more potent than kojic acid (80 ± 17 μg/mL), and they all had IC_50_ values bellow 1 μg/mL.

Similar to the report by Solimine, the polyphenol-enriched fraction of RODW, which contains flavonoid compounds, exhibits obvious tyrosinase inhibitory activity with an IC_50_ value of 0.41 ± 0.01 μg/mL [6]. Meanwhile, the tyrosinase inhibitory effects of RFCS is stonger than RODW from Pingyin [13]. The flavonoid content contributes to the overall tyrosinase inhibitory effect of RODW.

### Antimicrobial activities

The results of the antimicrobial activity studies of the different rose fractions, RFCS, and the standard antibiotics (tetracycline and hydrochloride) are presented in Table 4. *F. nucleatum* was most susceptible to RFCS and showed an MIC of 64 μg/mL and MBC of 250 μg/mL. The MIC values for RFCS against both *P. acnes* and *S. aureus* were 125 μg/mL. The MIC values against other bacteria were 250 μg/mL. The MIC and MBC or MFC values of the nine components of RFCS were determined to identify the constituents responsible for the antimicrobial effects of RFCS. *L. ivanovii* and *F. nucleatum* were found to be the most susceptible to α-bisabolol, and it showed MIC values of 8 μg/mL and MBC values of 32 μg/mL against these species (Table 4). After α-bisabolol, phenylethyl alcohol showed the lowest MIC and MBC or MFC values among all the constituents of RFCS. Overall, the volatile constituents played a more important role than the flavonoid compounds in the antimicrobial activity of RFCS.

**Table 4:** MIC and MBC or MFC of RFCS and different monomers against pathogenic bacteria (μg/mL)

Compound	*L. ivanovii*		*S. enteritidis enteritidis*		*S. aureus*		*E. coli*		*C. albicans*		*P. acnes*		*F. nucleatum*	
	MIC	MBC	MIC	MBC	MIC	MBC	MIC	MBC	MIC	MFC	MIC	MBC	MIC	MBC
RFCS	500	> 1000	500	> 1000	125	1000	500	1000	1000	> 1000	125	500	64	250
Linalool	500	> 1000	500	> 1000	250	1000	250	1000	250	1000	250	1000	250	1000
Phenylethyl alcohol	250	500	125	500	125	250	250	1000	250	500	125	500	8	32
Citronellol	250	500	250	1000	125	500	250	500	250	500	125	500	250	1000
α-bisabolol	8	32	500	> 1000	250	1000	500	1000	1000	> 1000	125	500	8	32
Hyperoside	250	500	250	500	250	1000	250	1000	250	1000	250	1000	250	500
Kaempferol-3-O-rutinosid	500	1000	500	> 1000	250	1000	500	> 1000	250	1000	500	1000	500	> 1000
Rutin	500	1000	125	500	250	500	250	1000	500	1000	125	250	62	500
Luteolin	500	1000	250	1000	500	> 1000	250	1000	250	500	250	500	125	500
Miconazole Nitrate	–	–	–	–	–	–	–	16	8	–	–	–	–	–
Hydrochloride tetracycline	< 0.1	< 0.1	8	16	16	16	16	–	–	16	8	16	2	2

Data are expressed as mean ± standard deviation of triplicate samples

*MIC* minimum inhibitory concentration, *MBC* minimum bactericidal concentration, *MFC* minimum Fungicidal concentration, *RFCS* rose flower cell sap, *L. ivanovii Listeria ivanovii*, *S. enteritidis subspecies enteritidis*: *Salmonella enteritidis enteritidis*, *S. aureus Staphylococcus aureus*, *E. coli Escherichia coli*, *C. albicans Candida albicans*, *P. acnes Propionibacterium acnes*, *F. nucleatum Fusobacterium nucleatum*

Previous investigations of the antimicrobial effects of the various fractions of rose have reported similar results [8, 28, 31]. The essential oil and various extracts of rose, including the aqueous extract, ethanol extract, chloroform extract, ethyl acetate fraction, and butanol fraction, exhibit broad-spectrum antimicrobial activities. With the exception of the ethyl acetate fraction, rose essential oil is comparatively more active against the tested bacteria [28]. The absolute and essential oils of rose contain high levels of polyphenols and phenylethyl alcohol, which result in outstanding antimicrobial properties [8]. Because the content of volatile oil in RODW is higher than RFCS, the antimicrobial effect of RODW better than RFCS [13]. The polyphenolic-enriched fraction from rugosa tea could inhibit *Escherichia coli* and *Pseudomonas aeruginosa* quorum sensing and biofilm formatiosignificancen [1]. The antimicrobial effects of some of the active ingredients of rose oil such as linalool, citronellol, and geraniol have been confirmed [32, 33]. To date, the antimicrobial activity of RFCS of *R. fenghua* has not been evaluated. This result explicitly supports the fact that high contents of phenylethyl alcohol and other volatile components contribute to the antimicrobial activities of RFCS [34].

## Conclusions

Our study demonstrated the strong antioxidant, antimicrobial, and tyrosinase inhibitory activities of RFCS. Due to the rose-like aroma of phenylethyl alcohol in combination with the tyrosinase inhibitory activities and antimicrobial effect against *S. enteritidis subspecies enteritidis*, *C. albicans*, and *P. acnes*, RFCS may be used as a natural skin-whitening and skin care additive in the cosmetics industry. Additionally, due to its antioxidant activities and antimicrobial effects against *L. ivanovii*, *S. subspecies*, *E. coli*, and *S. aureus*, RFCS can be used as a natural preservative and antimicrobial agent in the food and pharmaceutical industries.

## Abbreviations

BHI: Brain heart infusion
CDC: Anaerobic blood agar base medium
ESI: Electrospray ionization
GAM broth: Actinomycete broth medium
MBC: Minimum bactericidal concentration
MFC: Minimum fungicidal concentration
MIC: Minimum inhibitory concentration
MS: Mass spectrometer
RFCS: *Rosa rugosa Thunb. var. plena Regal* flower cell sap
RODW: Rose oil distillation wastewater
ROS: Reactive oxygen species
